# New Insights about How to Make an Intervention in Children and Adolescents with Metabolic Syndrome: Diet, Exercise vs. Changes in Body Composition. A Systematic Review of RCT

**DOI:** 10.3390/nu10070878

**Published:** 2018-07-06

**Authors:** Enrique Albert Pérez, Victoria Mateu Olivares, Rosa María Martínez-Espinosa, Mariola D Molina Vila, Manuel Reig García-Galbis

**Affiliations:** 1 Faculty of Health Sciences, University of Alicante, 03690 Alicante, Spain; ejalbertperez@gmail.com (E.A.P.); victoriamateu94@hotmail.es (V.M.O.); 2 Division of Biochemistry and Molecular Biology, Department of Agrochemistry and Biochemistry, Faculty of Sciences, University of Alicante, 03690 Alicante, Spain; rosa.martinez@ua.es; 3 Members of the Research Group of Applied Biochemistry (AppBiochem), Faculty of Sciences, University of Alicante, 03690 Alicante, Spain; mariola.molina@ua.es; 4 Department of Mathematics, Faculty of Sciences, University of Alicante, 03690 Alicante, Spain; 5 Department of Nutrition and Dietetics, Faculty of Health Sciences, University of Atacama, Avda Copayapu 2862, III Region, Copiapo 1530000, Chile

**Keywords:** metabolic syndrome, children, adolescents, diet, exercise, body composition, weight and fat

## Abstract

Objective: To record which interventions produce the greatest variations in body composition in patients ≤19 years old with metabolic syndrome (MS). Method: search dates between 2005 and 2017 in peer reviewed journals, following the PRISMA method (Preferred Reporting Items for Systematic reviews and Meta-Analyses). The selection criteria were: diagnostic for MS or at least a criterion for diagnosis; randomized clinical trials, ≤19 years of age; intervention programs that use diet and/or exercise as a tool (interventions showing an interest in body composition). Results: 1781 clinical trials were identified under these criteria but only 0.51% were included. The most frequent characteristics of the selected clinical trials were that they used multidisciplinary interventions and were carried out in America. The most utilized parameters were BMI (body mass index) in kg/m^2^ and BW (body weight) in kg. Conclusions: Most of the clinical trials included had been diagnosed through at least 2 diagnostic criteria for MS. Multidisciplinary interventions obtained greater changes in body composition in patients with MS. This change was especially prevalent in the combinations of dietary interventions and physical exercise. It is proposed to follow the guidelines proposed for patients who are overweight, obese, or have diabetes type 2, and extrapolate these strategies as recommendations for future clinical trials designed for patients with MS.

## 1. Introduction

### 1.1. Definitions

Diabetes (Diabetes mellitus: DM): serious medical condition in which body cannot control the amount of sugar in your blood.

Insulin resistance: is a pathology in which cells fail to respond normally to the hormone insulin. Insulin controls the concentrations of glucose in blood and it is produced by the pancreas when glucose starts to be released into the bloodstream from the digestion of carbohydrates (primarily) in the diet. Under normal conditions of insulin reactivity, this insulin response triggers glucose being taken into body cells, to be used for energy, and inhibits the body from using fat for energy, thereby causing the concentration of glucose in the blood to decrease. This, glucose concentration stays within the normal range even when a large amount of carbohydrates is consumed. During insulin resistance, excess glucose is not sufficiently absorbed by cells even in the presence of insulin, thereby causing an increase in the level of blood sugar. The insulin resistance syndrome (metabolic syndrome or syndrome X), and prediabetes are closely related and the show overlapping aspects.

Prediabetes (or “Pre-diabetic state): precursor stage before diabetes mellitus in which blood sugar is abnormally high. This stage is not a disease itself. Prediabetes is associated with obesity (especially abdominal or visceral obesity), dyslipidemia with high triglycerides and/or low HDL cholesterol, and hypertension. Thus, it is considered a metabolic diathesis or syndrome. Impaired fasting blood sugar and impaired glucose tolerance are two forms of prediabetes that are similar in clinical definition but are physiologically distinct. 

Impaired glucose intolerance (IGT): pre-diabetic state of hyperglycemia that is associated with insulin resistance and increased risk of cardiovascular pathology. IGT may precede type 2 diabetes mellitus by many years

Isolated impaired fasting glucose (IFG): pre-diabetic state in which the concentrations of sugar in blood during fasting are consistently above the normal range, but below the diagnostic cut-off for a formal diagnosis of diabetes mellitus. Together with impaired glucose tolerance, it is a sign of insulin resistance. In this manner, it is also one of the conditions associated with Metabolic Syndrome.

### 1.2. Noncommunicable Diseases (NCDs) 

Cardiovascular pathologies, cancers, chronic respiratory illnesses and diabetes are the primary causes of death around the world. More than 36 million people die annually from NCDs, which account for 63% of all deaths worldwide. These deaths are caused by poor diet, physical inactivity and the harmful use of alcohol and tobacco [1,2]. 

To prevent the development of these NCDs, the “Global Action Plan for the Prevention and Control of NCDs 2013–2020” was put into place by WHO (World Health Organization) and by the European health policy framework, Health 2020; thus, indicating a forward path for government and society. Among the voluntary global objectives, the following stand out: the reduction of widespread insufficient physical activity and the prevention of diabetes and obesity [1,2].

### 1.3. Metabolic Syndrome (MS): Concept and Prevalence (Table 1 and Table 2) 

Metabolic syndrome, also known as “Insulin Resistance Syndrome”, can be defined as a series of physiological, biochemical and metabolic factors that increase the risk for cardiovascular disease and type 2 diabetes (T2DM). These factors include insulin resistance, T2DM or glucose intolerance, hypertension and central obesity [3,4,5,6,7,8,9,10,11,12].

The concept of MS in the pediatric population is difficult to define due to the physiological changes throughout their growth and development, racial differences, and the lack of cardiovascular events [11,13]. The amount of clinical trials available on this age group is scarce, and therefore, a universal definition for children and adolescents does not exist thus far [11,13,14,15]. Since 2001, adaptations of the standardized adult MS definitions have been applied to the juvenile clinical trials [11,13,15,16].

These adaptations lead to excessive variety in diagnosis of MS. For example, the prevalence of MS in adolescents in the United States has been greater than 10% (2000–2010) [17]. However, depending on which diagnostic criteria is used, the prevalence can vary between 0.9, 3.8, 4.1, 10.5 and 11.4%. This wide variation can be directly attributed to the inconsistent terms and definition of MS in children and adolescents (Table 1 and Table 2) [18,19].

The diagnosis of obesity has evolved over time. The current definition of obesity based on weight and height cannot accurately identify all causes obesity-related risk of CVD (cardiovascular disease). People with a normal BMI (body mass index) and high content of BF (body fat) are at greater risk of metabolic disturbance, systemic inflammation and mortality. Thus, the metabolic alteration observed in individuals with normal weight metabolic obese can be only due to the increase of body adiposity not detected by the BMI [20].

### 1.4. Strategies for Intervention in Overweight, Obesity and T2DM

Thus far, specific guidelines for the treatments of MS have not been detailed. Therefore, evaluation and intervention guidelines on overweight, obesity and T2DM are presented (Table 3 and Table 4). 

Guides and/or algorithms for the management of the treatment of overweight, obesity and diabetes are technical reports supported by evidence. They contain an outline of interventions, indicating what must be done on these pathologies. Table 3 and Table 4 summarize some guidelines, however, there are other guides not mentioned in this work [32,33,34,35,36,37]. Most of these guides are revised to evaluate the degree of evidence for each recommendation (Table 3). Thus, these guides show a consensus in the evidence regarding dietary techniques and physical exercise (Table 3). However, there are differences between the consensus established between these guides in terms of energy restriction and the recommendations related to the percentage of intake of macronutrients (Table 4). The consensus was obtained from clinical trials where the authors observed a decrease of BMI and/or body weight. Probably, this controversy could disappear if guides and/or algorithms record those clinical trials that consider the BMI and/or weight along with other anthropometric parameters, such as body fat and fat-free mass [38,39,40,41].

### 1.5. Changes in Body Composition Andmetabolic Abnormalities

At present the metabolic changes are being considered as a cardiometabolic syndrome, which is a set of various risk factors such as abdominal obesity, hypertension or hypertension, dyslipidemia, and prediabetes [42,43]. In response to this syndrome or metabolic alterations, the need has emerged to use better tools to monitor the patterns of individual growth, assess body composition in risk and identify those who are at increased risk of developing metabolic components of the disease. The risk assessment of this pathology should be evaluated beyond the capacity of the BMI and/or body weight, hence the need arises for other anthropometric parameters, such as the percentage of body fat, fat-free mass and/or skeletal muscle mass [40,41].

Current evidence suggests that the intervention of physical exercise in adolescents with overweight and obesity improves body composition, changes body fat, and therefore could improve some cardio-metabolic factors [44]. In the lifestyle interventions, the authors of these studies relate the changes in body weight with the cardio-metabolic results [45]. The most traditional dietary patterns, including the Mediterranean diet, are associated with better metabolic profiles [46].

### 1.6. Use of Pharmacology in the Interventions of Changes Body Composition

The advantage of using medication in interventions for the management of weight loss in patients aged 2 to 18 years is not yet clear [32,47]. In relation to the use of drugs in children and adolescents with prediabetes in 2017, clinical guidelines from the Endocrine Society recommend that pediatricians abstain from prescribing pharmacotherapy, including metformin [48]. However, there are other bibliographic sources that recommend its use [30,49]. The American Diabetes Association recognizes insulin and metformin as treatment for T2DM [50].

### 1.7. Theoretical Framework and Purpose of the Review

The interest and novelty of this systematic review are justified by the following premises:

1. Due to the prevalence observed in children and adolescents with MS [17,18,19].
2. The search for which dietary intervention and physical exercise obtains greater changes in body composition in children and adolescents with MS, as described by the overweight, obesity and T2DM guidelines [27,29,30].
3. The relationship between the changes in body composition and cardio-metabolic factors [44,45].
4. Adhering to the WHO Global Action Plan in the reduction [1,2], which is focused on the factors related to the diagnosis of MS [3,4].

The principal objective is to record which interventions produce the greatest variations in body composition in patients ≤19 years old with MS. 

The secondary objectives are: (a) to identify which interventions, produce the greatest changes in body composition in patients ≤19 years of age with MS, either exclusive or multidisciplinary; (b) to indicate which anthropometric parameters and units of measurement were the most used to record changes in body composition in patients ≤19 years old with MS.

## 2. Method

### 2.1. Selection Criteria of the Clinical Trials and Search Strategy 

**Figure 1:**
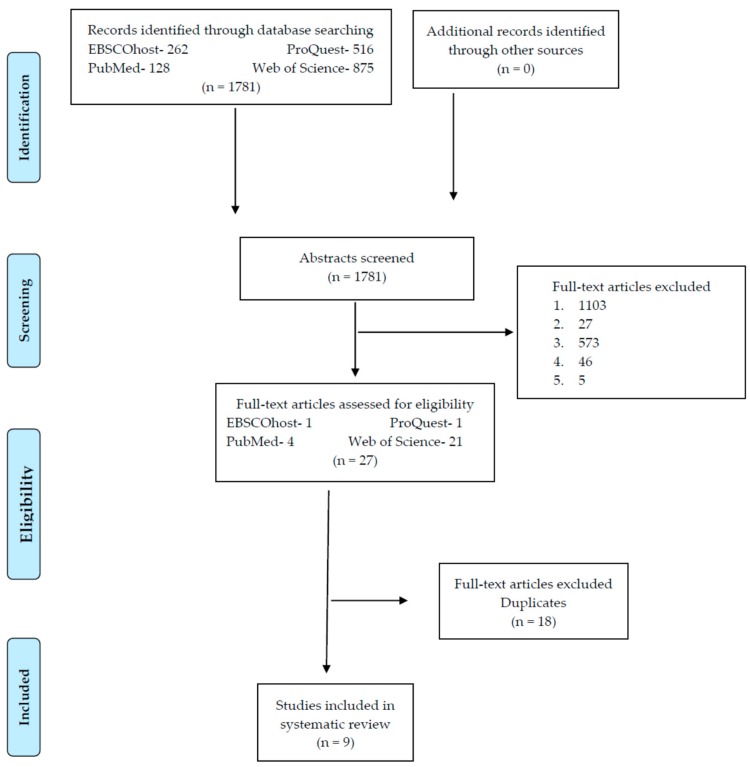
Flow chart of the screening process for the selection of included clinical trials [52,53,54].

The selection criteria were: randomized clinical trials in humans; patients ≤19 years old [51] that had been diagnosed with MS and otherwise, should have been diagnosed using at least two diagnostic criteria (T2DM, high blood pressure, insulin resistance, hyperinsulinemia, hyperinsulinism, hyperglycemia, dislipemia, glucose intolerance and/or prediabetes) (Table 1 and Table 2); intervention programs including diet, physical exercise and/or modifications in the style of life as treatment as well as the magnitude of changes in body composition; clinical trials published between 2005 and 2017 in scientific journals and in Spanish and English (Table 6). The exclusion criteria are detailed in the flowchart, Figure 1.

This observational investigation was based upon a systematic review following the recommended guidelines of PRISMA (Preferred Reporting Items for Systematic reviews and Meta-Analyses, Table S1) [52,53,54] and it used the information retrieval system “the Boolean Model” [55]. All data used were collected from the following databases: PubMed, EBSCOhost, ProQuest and Web of Science in articles of original clinical trials. The key words were: metabolic syndrome, children, teens, pediatrics, diet and exercise; the database “MeSH” (Medical Subject Headings) (Table 5 and Figure 1).

Using EBSCOhost, all of the databases that included the following options were selected: “AB Summary” and “academic publications” in types of publications; in PubMed an advanced search was used and the following options were selected: “Title/Abstract” and type of article was “Clinical Trial”; in ProQuest an advanced search was used and the following options were selected: “scientific journals” in type of source, “Primary article” in type of document and “Abstract”; in Web of Science a basic search was used and the following options were selected: “Clinical Trial” in types of documents and “theme”.

The primary research question was: which are the clinical trials reporting the greatest variations in body composition, in patients <19 years old with MS?

### 2.2. Data Extraction, Synthesis of Results and Risk of Information Loss 

Six tables and 3 figures were designed to execute this systematic review. Table 1, Table 2, Table 3 and Table 4 identify the most relevant background information for the design of the theoretical framework. The flowchart displays the selection process of the clinical trials included (Figure 1). 

Table 5 reflects which strategies and databases were utilized. Table 6 records the most relevant details about the clinical trials included and was ordered from the simplest intervention techniques (exclusive) to the most difficult (multidisciplinary). The searches were made independently (one for each of the authors). Therefore, there were five versions of Table 6, all of them including as much information as possible from the trials included. The rest of the tables were performed jointly. 

As for the risk of information loss, only one clinical trial was found where the authors diagnosed MS (Table 6) [56]. Heterogeneous designs were identified in the selected clinical trials. The variations found were: the use of great diversity in the MS diagnostic criteria (Table 1), signs and/or pathologies prior to having MS (Table 2); disparity in the number of patients and their age; temporal duration; diversity in the intervention strategies used; diversity in the anthropometric parameters and units with which the changes of body composition are expressed (Table 6).

## 3. Results

### 3.1. Search Characteristics and Types of Identified Interventions

Of the 1781 clinical trials identified, only 0.51% were included (Figure 1 and Table 5). The most useful search engine was Web of Science and the most effective search strategy was the fourth (Table 5).

From the most frequently observed characteristics in the randomized clinical trials (Table 6), we can highlight: completed intervention programs, 55.56% of which had a duration of 6 months (3 to 18-month range). Six of the studies had a female presence that was slightly larger than the male. The places of origin were: America (66.67%), Oceania (11.11%) and Europe (22.22%). The age of participants ranged from 4 to 19 years of age, while the simple size included between 25 to 150 participants (Median, 58 individuals). The pathology with the most incidence was insulin resistance (66.66%).

All the studies show two intervention groups. The observed intervention modalities were: diet, physical exercise, psychology, and pharmacology; Multidisciplinary interventions were the most frequent at 88.89% (Table 6).

Legend of exclusion criteria (Figure 1):

1. Include a part or a sample without the objective pathology of the systematic revision
2. Dietary and physical exercise interventions or education to changes body composition not defined
3. No comparison or analysis of the anthropometric parameter of interest, before or after intervention
4. Include adult sample (older than 18 years old)
5. Sample not human

### 3.2. Variations in Body Composition; Exclusive vs. Multidisciplinary Intervention (Table 6)

In response to the principal objective and research question of this systematic review, the greatest changes in body composition from the following clinical trials were reported. The Mello MT clinical trial showed the greatest changes in body composition, in BW (15.45 ± 6.95 kg, which represents a change with respect to the medium base weight 14.27%), in BF, in kg and in % of BW, of 17.34 ± 6.5 kg and 11.42 ± 6.10%, respectively in BMI of 5.54 ± 2.41 kg/m^2^ and in WC of 17.06 ± 11.38 cm. It was a multidisciplinary intervention of 30 total patients (15 per group) without the use of medication that combines dietary intervention, physical exercise, and both group and individual psychology from a medical professional. The intervention that provokes these changes is based on a balanced diet with energy expenditure of low physical activity, despite the completed physical exercise (which includes both aerobic and resistance exercises), one hour of group psychology, and monthly medical follow-ups. This clinical trial is also the only one to present data close to the lean mass (LM). The group which performed both aerobic and resistance exercises demonstrated greater growth in the body composition of LM (2.31 ± 5.22 kg) [56].

The Gomez-Diaz clinical trial was the only trial to perform the statistical study of the percentage changes of BW, obtaining a change of 5.86% in the group treated with metformin (with a range of 2.0–19%). This trial was treated as a multidisciplinary intervention based on the use of a balanced diet, a normocaloric diet, the inclusion of moderate physical exercise and the use of metformin [60]. None of the trials perform a study on the percent changes of other parameters.

The Van der Aa. MP clinical trial is the only one which reports information close to the variation in body composition in FFM. It was a multidisciplinary intervention based on the inclusion of physical exercise, carried out twice a week with supervision of a physical therapist, and with training courses. It is the second intervention group to not use metformin [58]. The median range of growth was 4.5 kg with an interquartile range from 1.3 to 11.6 kg in the group that did not use metformin, versus the group that did. 

In response to the secondary objective of this review, the multidisciplinary interventions are the ones with the most variation in body composition, especially with the combination of dietary intervention and physical exercise [56,58,60].

### 3.3. Anthropometric Parameters and Units of Measurement to Express the Changes in Body Composition

**Figure 2:**
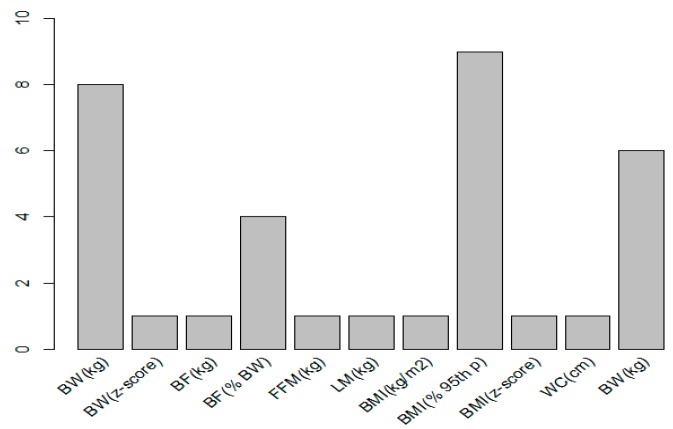
Number of articles within the study of the corresponding parameter and unit.

Regarding the final secondary objective of this review, the anthropometric parameters and units of measurement were BMI in kg/m^2^ (analyzed by all articles) and BW in kg (8 out of 9 articles). The percentage of use of the rest of the parameters and units that appear in Table 6, are reflected in Figure 2. 

In Table 6, the variations of body composition are compiled from each of the 2 intervention groups for each studied variable. It shows if significant differences are present in the changes within each intervention group, as well as between them. 

With respect to the sole article using exclusive interventions, it studies BW, BF, BMI and WC, but only reports a statistically significant (*p*-value < 0.05) change in WC between the intervention groups (−9.1 ± 4.8 cm in the group with a low insulin response diet versus −6.6 ± 4.6 cm in the group with conventional diet). It does not report information about differences within groups [57].

Concerning the multidisciplinary intervention trials, 87.5% of them included the study of BW changes in kg, in *z*-score and/or relative units. Three of them do not report any information about the differences within intervention groups (final vs. baseline levels). Regarding the differences in BW between intervention groups, five present the differences and are statistically significant in four of the cases. 

Regarding BMI, all the papers include the variable for their study in some of the units. All of them study the parameter in absolute units, but only five report significant differences between the intervention groups. 

The loss in WC is collected in five of the eight works, registering a statistically significant change between intervention groups in two of them. 

As for the rest of the units and parameters, in each case there is only one work that presents its study. 

**Figure 3:**
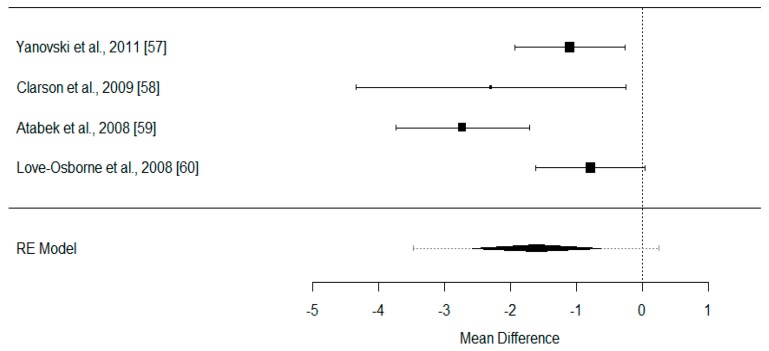
Confidence interval (95%) of the difference in BMI (kg/m^2^) between intervention groups.

Figure 3 represents the forest plot of the four works that present data for the calculation of the variable BMI in kg/m^2^. All of them are studies where the difference between the two intervention groups lies on the use or nonuse of metformin. However, it should be noted that they present a high degree of heterogeneity (*I*^2^ = 70.46%).

## 4. Discussion

### 4.1. Changes in Body Composition. Comparison between This Work and Other Reviews and/or Meta-Analyses 

The most important variations in the parameters and units used to analyze body composition are discussed in the following. Comparisons between the main conclusion from this work, as well as from other reviews, are also included (Table 6).

Until now, reviews and/or meta-analyses that analyze the parameters and units for expressing body composition in patients with MS or T2DM have not been found. Thus, the comparisons were made with the reviews and/or meta-analyses of children and adolescents with overweight, obesity, insulin-resistance or prediabetes.

In this literary search, it has not been possible to find reviews or meta-analyses that present a clinical trial with a greater variation in body composition that the clinical trials analyzed in this systematic review [44,65,66,67,68,69]. 

Most reviews and/or meta-analyses that have been analyzed do not consider the variation in body composition that occurs when diet and physical exercise are used as treatment tools in these patients [7,25,70]. It has been demonstrated that the changes in body composition produce favorable changes in the metabolic illness risk factors in children, adolescents and adults [71], which is why further research in this area is recommended. 

### 4.2. Practical Recommendations for the Design of Future Clinical Trials of Patients with Overweight, Obesity, T2DM and MS (Table 3 and Table 4)

Anthropometric parameters and units of measurement express the changes in body composition through the changes of some parameters (BW, BF, BMI and WC) and increase of other parameters (FFM and LM). Due to the heterogeneity in the design of clinical trials analyzed here, and considering the main conclusion from this research, the authors propose the following indications for future trials:

1. Follow the guidelines for the design of randomized trial reports [72] and other randomized guides [73].
2. The use of the percentage in both the BW as in BF is recommended (in parameters as well as in the units used), especially when the sample is composed of individuals of different ages and/or genders [38,41,74,75,76].
3. The BF is an indicator of the quality of the changes obtained in body composition [38,74,75,77].
4. Set the limit at 5% changes of BF to evaluate the effectiveness of the intervention [38,68,74,78].
5. The changes in body composition (BW, BF and BMI) not only occurs with the assistance of the patient, but also the consultation as can be seen in Table 4 [74].
6. Based on the lifestyle changes in weight loss programs, the following results were obtained [32]: (6a) One contact with the patient in ≥26 h, demonstrating small reductions in weight excess in overweight and obese patients, without evidence of harm. (6b) One contact with the patient in ≥52 h, demonstrating an improved blood pressure and other cardio-metabolic improvements.
7. In the dietary intervention, at the stage of changes in body composition, the type of energy restriction must be selected depending on the degree of excess weight (Table 3 and Table 4). Besides, the following recommendations must also be considered: (7a) an adaptation in the daily planning of the energy density and macronutrients if the patient completes the physical exercises regularly, especially the intake of complex carbohydrates [79]. (7b) be especially cautious regarding the planning of the carbohydrate intake guidelines for the days where physical exercise is completed [80].
8. In dietary interventions a period of treatment up to 6 months is recommended, because this is the time required to reach the maximum average WL and the BF (%) [38,74,81]. Recommendations for the reduction of body composition are in Table 3 of this review.
9. Table 3 and Table 4 summarize the strategies that must be shown by an intervention of physical exercise in patients looking for changes of body composition (BW, BF, BMI and WC). In addition, according to the American College of Sports Medicine (ACSM) in clinical trials including physical exercise, the following aspects should be recorded [82]: (9a) Cardiorespiratory fitness exercise of resistance: the frequency (days per week), intensity (mild, moderate or vigorous), time (duration), type, volume (the distance travelled or the expenditure of energy that causes), pattern (one or more than one session for day) and progression (in volume of exercise adjusted to the duration, the frequency and/or intensity). (9b) Resistance exercise: frequency (each muscle group should be trained 2–3 times per week), intensity (mild, moderate or vigorous in function of the maximum repetitions and on the weight lifted), time (to be determined), the type (depending on the muscle group involved and the weight lifted); repetitions, session or patterns (rest intervals of 2–3 min between each set of repetitions; it is recommended ≥48 h between sessions for any muscle group) and progression (gradual increase of greater resistance, and/or more replicates per set).
10. In the article by Pieles GE and colleagues (Table 1 and Figure 1), it is shown that progress has been made in the recommendations for children and adolescents. However, a more accurate vision should be taken as indicated by ACSM both theoretically and practically to be able to prove effectiveness [82,83]. For example, in the maintenance and/or reduction of body composition in patients with overweight, obesity, T2DM and MS, the amount of time they can be seated in front of the television, must be limited and relative to their ages (Table 4). However, occasionally this recommendation is not met [84].
11. Mark the objective of the changes in body composition, around a 5, 10 or 15% changes of body weight or body fat, without necessity to set an ideal BMI [85,86]. One should be cautious when it comes to not regaining the weight and/or fat [87] and be more tolerant of variations in body composition [85].
12. Dietician-nutritionists must be involved in the design of the dietary intervention [81,88,89,90].
13. A healthcare professional must have a role as manager who informs and educates about the most effective options in the process of decreasing body composition [90,91].

Therefore, it is recommended to follow the recommendations for the design of future clinical trials of patients with MS. In addition, these recommendations may also be used for patients with overweight, obesity, pre-diabetes, insulin resistance and T2DM. 

### 4.3. Limitations and Strength of the Systematic Review

The strengths of this systematic review are:

1. The range of search dates of this systematic review, having found 1781 clinical trials from 2005–2017.
2. The following contributions to the review of this subject: (2a) The MS definitions and diagnostic criteria (Table 1 and Table 2). (2b) A synthesis of guides about the treatment of overweight, obesity, and T2DM (Table 3 and Table 4) together with the development of practical recommendations for the design of future clinical trials related to MS in children and adolescents. This is due to the lack of guidelines and consensus on MS in children and adolescents.
3. The trial has been carried out and overseen by professionals versed in dietary intervention and physical exercise for patients with overweight, obesity, and T2DM [38,39,74,77,90,92,93].

The limitations of this systematic review were previously discussed in the theoretical framework and in the section on risk methodology of information loss.

## 5. Conclusions

With the increasing prevalence of MS, the lack of unification in the accepted diagnostic criteria of MS makes it difficult to determine the prevalence of this syndrome and its use as a diagnostic criterion.

In this systematic review, most of the included clinical trials with children and adolescents have been diagnosed by at least one MS diagnostic criterion, which implies the lack of use of the MS definitions. 

The relationship between the changes in body composition (changes of some parameters such as BW, BF, BMI and WC; increase of other parameters such as FFM and LM) and metabolic abnormalities of the MS, make it advisable to increase the research in this area.

The parameters and units showing the highest changes in body composition were BW (kg) and BMI (kg/m^2^).

The evidence is still not clear as to the use of medication in the intervention programs for the changes of body weight in children and adolescents. 

It is proposed to follow the guidelines proposed for patients with overweight, obesity and T2DM (Table 3 and Table 4) and extrapolate these strategies as recommendations to the future clinical trials designed in patients with MS.

## Figures and Tables

**Table 1:** Diagnosis of metabolic syndrome in children and adolescents.

		AHA Criteria [21]	IDF Criteria			WHO Criteria	NCEP ATP III Criteria
**Necessary components for the MS diagnosis**		3 of the 5 must be present	Central obesity and 2 of 4 other components must be present [21]			At least 3 or hyperinsulinemia and at least 2 must be present [22]	At least 3 must be present [23]
**Age (years)**		12–19	6–9 [21]	10–15 [21] 10–16 [23]	>15 [21] ≥16 [24]	ND	ND
**Essential criteria**		ND	ND	ND	ND	Insulin resistance [23]	None [23]
**Waist circumference**		WC ≥ 90th percentile for age, sex and race/ethnicity	WC ≥ 90th percentile for age (MS as entity is not diagnosed) [21]	WC ≥ 90th percentile [23] or adult cut-off if lower [21]	WC ≥ 90 cm in boys and ≥80 cm in girls [24] WC ≥ 94 cm in boys and ≥80 cm in girls [21]	Waist-to-hip ratio > 0.9 in boys and >0.85 in girls [23] BMI ≥ 75/85/95th percentile by age, sex [22]	WC ≥102 cm in boys and ≥88 cm in girls [23] WC > 90th percentile or BMI ≥ 97th percentile [22] WC > 75th percentile for age and sex [24]
**BMI**		ND	ND	ND	ND	>30 kg/m^2^ [23]	ND
**Blood pressure**		≥90th percentile for age, sex, and height	ND	SBP ≥ 130 mmHg [23] SBP ≥ 130 mmHg or DBP ≥ 85 mmHg [24]	SBP ≥ 130 mmHg or DBP ≥85 mmHg [24] or treatment of previously diagnosed hypertension [21]	SBP ≥ 140 mmHg [23]	SBP ≥ 130 mmHg [23] SBP > 90th percentile for age and sex [24]
**Dyslipidemia**	**Triglyceride**	≥1.23 mmol/L (≥110 mg/dL)	ND	≥1.7 mmol/L (≥150 mg/dL) [23]	≥1.7 mmol/L (≥150 mg/dL) [24] or specific treatment for high triglycerides [21]	≥1.7 mmol/L (≥150 mg/dL) [23]	≥1.7 mmol/L (≥150 mg/dL) [23] ≥100 mg/dL [24]
	**HDL-C**	≤10th percentile for race and sex [21]	ND	<1.03 mmol/L (<40 mg/dL) [23]	<1.03 mmol/L (<40 mg/dL) in boys and <1.29 mmol/L (<50 mg/dL) in girls [24] or specific treatment for low HDL-C [21]	<0.91 mmol/L in boys <1.0 mmol/L in girls [23]	<1.0 mmol/L [23] 500 mg/dL, except boys from 15 to 18 years, whose cutoff point was <45 mg/dL [24]
**Glucose**		Fasting glucose ≥5.6 mmol/L (≥100 mg/dL) [21]	ND	Fasting glucose ≥5.6 mmol/L (≥100 mg/dL) [23]	Fasting glucose ≥5.6 mmol/L (≥100 mg/dL) [24] or known T2DM [21]	Insulin resistance or diabetes [23] Fasting glucose ≥ 6.1 mmol/L (≥110 mg/dL) or ≥80/90th percentile by age, sex or diabetes [22]	Fasting glucose ≥6.1 mmol/L (≥110 mg/dL) [23] Fasting glucose ≥5.6 or 6.1 mmol/L (≥100 or 110 mg/dL) or 2 h glucose ≥140 mg/dL [22]
**Insulin**		ND	ND	ND	ND	Insulin resistance [23]	ND

AHA: American Heart Association; BMI: body mass index; cm: centimeters; DBP: diastolic blood pressure; HDL-C: high-density lipoprotein cholesterol (lipoproteins that carry cholesterol from the tissues of the body to the liver); IDF: International Diabetes Federation; MS: metabolic syndrome; NCEP ATP III: National Cholesterol Education Program’s Adult Treatment Panel; ND: not declared; SBP: systolic blood pressure; T2DM: type 2 diabetes mellitus (type of glycerol that belongs to the family of lipids, in mammals it is transported throughout the body while supplying energy or is stored as fat, for long periods; WC: waist circumference; WHO: World Health Organization.

**Table 2:** Diagnostic criteria for prediabetes, impaired glucose tolerance and type 2 diabetes mellitus in children and adolescents.

			WHO Criteria	ADA Criteria
**Prediabetes [25]**	Glucose	Fasting plasma glucose	110–125 mg/dL (6.1–6.9 mmol/L)	100–125 mg/dL (5.6–6.9 mmol/L)
		Random Plasma Glucose	ND	Not applicable
		2-h plasma glucose (OGTT)	140–200 mg/dL (7.8–11.0 mmol/L)	140–200 mg/dL (7.8–11.0 mmol/L)
	Hemoglobin A1c		ND	5.7–6.4%
**Impaired glucose tolerance [26]**	Glucose	2-h plasma glucose (OGTT)	ND	140–199 mg/dL (7.8–11.0 mmol/L)
**Type 2 Diabetes Mellitus [25,26]**	Glucose	Fasting plasma glucose	ND	≥126 mg/dL (7.0 mmol/L)
		Random Plasma Glucose	ND	≥200 mg/dL (11.1 mmol/L)
		2-h plasma glucose (OGTT)	ND	≥200 mg/dL (11.1 mmol/L)
	Hemoglobin A1c		ND	≥6.5%

ADA: American Diabetes Association; Fasting plasma glucose: fasting for at least 8 h with no calorie intake; OGTT (2-h plasma glucose): OGTT using a load of glucose 1.75 g/kg of body weight, with a maximum of 75 g; Random plasma glucose: In patients with hyperglycemic crises or classic symptoms of hyperglycemia (e.g., polyuria, polydipsia); diabetes: In the absence of unequivocal hyperglycemia, diagnosis is confirmed if two different tests are above threshold or a single test is above threshold twice; A1c: glycosylated hemoglobin; OGTT: Oral Glucose Tolerance Test; ND: Not Declared; WHO: World Health Organization.

**Table 3:** Guidelines and consensus on the treatment of overweight and obesity: children and adolescents *.

		Author	Recommendations in Dietary Intervention and Exercise
Overweight and obesity	AND	[27]	**Intervention:** divided into three levels: primary, secondary and tertiary prevention Evidence: 2009 Academy of Nutrition and Dietetics (Figure 1) [28]
	ICSI	[29]	**Intervention:** during the day, diet and physical activity. It identifies 4 levels of intervention in patients with BMI ≥ 85th percentile: prevention, structures weight management, integral multidisciplinary intervention, tertiary intervention **Dietary intervention:** the consumption of a diet with very low energy density **BW**: age, 2–11 years = 1 lb or 0.45 kg mo^−1^; age, 12–18 years = 2 lb or 0.91 kg wk^−1^ Evidence: [29]
T2DM		[30]	**Dietary intervention:** Interventions to reduce pediatric obesity should be multicomponent and include diet, physical activity, nutritional consulting and require participation of the parents or guardians. A nutritional prescription should be formulated as part of the dietary intervention in a multi component pediatric weight control program. The dietary factors that can be associated with the greatest risk for obesity are increasing the total amount of fats in diet as well as increasing the intake of beverages. The dietary factors that can be associated with the least risk for obesity is the increase of fruits and vegetables. The familiar dietary behaviors that are associated with the greatest risk for pediatric obesity are the parental restriction of healthy foods, the consumption of food outside the house (e.g., fast food), the large portion sizes of meals and the skipping of breakfast. Evidence: does not use the system of degrees of evidence

* Extensive information is given in Table 4; AND: Academy of Nutrition and Dietetics; ICSI: Health Care Guideline; VLCD: very low energy density diets; day (d); kilograms (Kg); minutes (min); month (mo); week (wk); pounds (lb).

**Table 4:** Intervention strategies for the reduction of body composition in overweight, obesity and T2DM: children and adolescents.

**Dietary Intervention**		
**Energy restriction**	**Overweight and obesity**	1000 a 2000 Kcal day^−1^ [27]
	**T2DM**	≥1200 Kcal day^−1^ in ages between 6 and 12 years old [30]
**VLCD**	**Overweight and obesity**	≤1.000 Kcal day^−1^ ó 600 a 800 Kcal day^−1^ (PSMF) [27]
	**T2DM**	≥900 Kcal day^−1^ in ages between 6 and 12 years old [30]
**Macronutrients and diets**		Different quantities of macronutrients (carbohydrates, proteins and fats) and different types of diets; PSMF (10–20 weeks), proteins (1.5 to 2.0 g kg^−1^ to reach the optimum body weight), carbohydrates (20–25 g day^−1^), water and other liquids without calories (2 L day^−1^), daily multivitamin supplements, balanced diet (for 10 weeks) [27]
**Physical exercise**		
**Overweight and obesity**		≤2 years old should not watch television, supervised free play is encouraged; 4 to 6 years old, up to 120 min of moderate to rigorous physical activity (MVPA) each day, 60 min in structured activity and 60 min of free play; ≥10 years old, at least ≥60 min day^−1^ of physical activity which should consist primarily of MVPA. In adolescents, promote and incorporate more complex and personalized activities [29]
**T2DM**		Children and adolescents with T2DM should practice moderate to vigorous physical activity for at least 60 min day^−1^ a day [27,31] Limited television time, to less than 2 h per day [27] Evidence grade D: expert opinions and evidence from metabolic syndrome and obesity studies. Prevalence of benefits over the harms.

T1DM: Diabetes mellitus type 1; PSMF: high protein diet.

**Table 5:** Search strategies of identified and included clinical trials, dates: 2005–2017.

Search strategy	EBSCOhost Identified/Included	ProQuest Identified/Included	PubMed Identified/Included	Web of Science Identified/Included
“metabolic syndrome” AND “children” OR “teens” OR “pediatrics” AND “diet” OR “dietary treatment” OR “feeding” AND “nutrition” OR “nutritional counseling” OR “lifestyle”	12/0	25/0	3/0	32/1
“metabolic syndrome” AND “children” OR “teens” OR “pediatrics” AND “exercise” OR “physical activity” OR “sport” OR “weightlifting”	54/0	90/0	13/0	44/2
“metabolic syndrome” AND “children” OR “teens” OR “pediatrics” AND “weight loss” OR “weight reduction” OR “fat loss” OR “fat reduction”	12/0	27/0	8/0	277/3
“type II diabetes” OR “insulin resistance” OR “hyperinsulinism” OR “hyperinsulinaemia” OR “hyperglycemia” OR “dyslipidemia” OR “prediabetes” AND “children” OR “teens” OR “pediatrics” AND “weight loss” OR “weight reduction” OR “fat loss” OR “fat reduction”	29/1	75/1	25/2	88/7
“type II diabetes” OR “insulin resistance” OR “hyperinsulinism” OR “hyperinsulinaemia” OR “hyperglycemia” OR “dyslipidemia” OR “prediabetes” AND “children” OR “teens” OR “pediatrics” AND “diet” OR “dietary treatment” OR “feeding” AND “nutrition” OR “nutritional counseling” OR “lifestyle”	23/0	55/0	13/1	78/2
“type II diabetes” OR “insulin resistance” OR “hyperinsulinism” OR “hyperinsulinaemia” OR “hyperglycemia” OR “dyslipidemia” OR “prediabetes” AND “children” OR “teens” OR “pediatrics” AND “exercise” OR “physical activity” OR “sport” OR “weightlifting”	114/0	200/0	65/1	135/6
“metabolic syndrome” AND “hypertension” OR “high blood pressure” AND “children” OR “teens” OR “pediatrics” AND “weight loss” OR “weight reduction” OR “fat loss” OR “fat reduction”	3/0	6/0	0/0	45/0
“metabolic syndrome” AND “hypertension” OR “high blood pressure” AND “children” OR “teens” OR “pediatrics” AND “diet” OR “dietary treatment” OR “feeding” AND “nutrition” OR “nutritional counseling” OR “lifestyle”	4/0	13/0	0/0	40/0
“metabolic syndrome” AND “hypertension” OR “high blood pressure” AND “children” OR “teens” OR “pediatrics” AND “exercise” OR “physical activity” OR “sport” OR “weightlifting”	11/0	25/0	1/0	136/0

**Table 6:** Characteristics of included randomized trials in children and adolescents.

Author [56,57,58,59,60,61,62,63,64]	Sample/Diagnostic Criteria	Duration (Months)	Intervention and Comparative Statistical Analysis of the Body Composition	BW (kg or *z*-Score/%)	BF (kg or % of BW)	FFM (kg/)	LM (kg)	BMI (kg/m^2^ or % of 95th Percentile or *z*-Score)	WC (cm)	Changes in Body Composition Mean ± sd or Mean ± (SE) or Mean (CI, 95%)
Armeno et al., 2011 [57]	*n* = 86 IG1: 47 IG2: 39 Girls: 58% Age: 11–19 years old Population: South America (Argentina) Obesity and Insulin Resistance, source of diagnostic criteria: 95th Percentile /NE	4	**Dietary intervention** Within groups: IG1 (low insulin response diet) IG2 (conventional diet) Between groups	**YES (kg and *z*-score)** NE NE NS	**YES (kg)** NE NE NE	NO	NO	**YES (kg/m^2^ and*z*-score)** NE NE NS	**YES** NE NE *p* < 0.05	**BW**: IG1: 8.9 kg IG2: −6.4 kg IG1: −0.53 ± 0.5 (*z*-score) IG2: −0.54 ± 0.4 (*z*-score) **BF**: IG1: −5.12 kg IG2: NE **BMI**: IG1: −3.9 kg/m^2^ IG2: −2.9 kg/m^2^ IG1: −0.35 ± 0.2 (*z*-score) IG2: −0.36 ± 0.2 (*z*-score) **WC**: IG1: −9.1 ± 4.8 cm IG2: −6.6 ± 4.6 cm
Van der Aa et al., 2016 [58]	*n* = 42 IG1: 23 IG2: 19 Girls: 66% Age: 10–16 years old Population: Europe (The Netherlands) Obesity and Insulin Resistance, source of diagnostic criteria: NE/NE	18	**Physical exercise intervention, pharmacology** Within groups: IG1 (metformin) IG2 (placebo) Between groups	**YES (kg)** NE NE NE	**YES (kg and % of BW)** NE NE *p* < 0.05/NS	**YES** NE NE *p* < 0.05	NO	**YES (kg/m^2^)** NE NE *p* < 0.05	**YES** NE NE NE	**BW ^(3)^:** IG1: 1.6 kg (−4.2, 5.9) IG2: 12 kg (2.7, 17) **BF ^(3)^**: IG1: −0.2 kg (−5.2, 2.1) IG2: 2 kg (1.2, 6.4) IG1: −3.1% (−4.8, 0.3) IG2: −0.8% (−3.2, 1.6) **FFM ^(3)^:** IG1: 2.0 kg (−0.1, 4) IG2: 4.5 kg (1.3, 11.6) **BMI ^(3)^**: IG1: 0.2 kg/m^2^ (−2.9, +1.3) IG2: 1.2 kg/m^2^ (−0.3, 2.4) **WC ^(3)^**: IG1 and IG2 NE (cm)
Garnett et al., 2013 [59]	*n* = 111 IG1: 55 IG2: 56 Girls: 61% Age: 10–17 years old Population: Oceania (Australia) Overweight and Obesity/Prediabetes and/or Insulin Resistance, source of diagnostic criteria: International Obesity Task Force/ADA/NE	6	**Dietary and physical exercise intervention, pharmacology** Within groups: IG1 (high CH diet) IG2 (low CH diet) Between groups	**YES (kg)** NE NE NE	NO	NO	NO	**YES (% of 95th percentile)** *p* < 0.05 *p* < 0.05 NS	NO	**BW**: Total: −3.7 kg (median) **BMI**: Total NE % 95th percentile
Gómez-Díaz et al., 2012 [60]	*n* = 52 IG1: 28 IG2: 24 Girls: 56% Age: 4–17 years old Population: North America (Mexico) Glucose Intolerance, source of diagnostic criteria: ADA	3	**Dietary intervention, physical exercise education, pharmacology** Within groups: IG1 (metformin) IG2 (placebo) Between groups	**YES (kg) /YES** *p* < 0.05 *p* < 0.05 NS/ *p* < 0.05	NO	NO	NO	**YES (kg/m^2^)** *p* < 0.05 *p* < 0.05 NS	**YES** *p* < 0.05 *p* < 0.05 NS	**BW**: IG1: −2.7 kg IG1: −5.86% IG2: −1.6 kg IG2: −2.75% **BMI**: IG1: −4.3 kg/m^2^ IG2: −1.0 kg/m^2^ **WC**: IG1: −9.3 cm IG2: −5.7 cm
de Mello et al., 2011 [56]	*n* = 30 IG1: 15 IG2: 15 Boys: 67% Age: 15–19 years old Population: South America (Brazil) Metabolic Syndrome/ Obesity, source of diagnostic criteria: IDF/>95th Percentile	12	**Physical exercise, dietary and psychological intervention, clinical therapy** Within groups: IG1 (aerobic training) IG2 (aerobic plus resistance training) Between groups	**YES (kg)** *p* < 0.05 *p* < 0.05 *p* < 0.05	**YES (kg and % of BW)** *p* < 0.05 *p* < 0.05 *p* < 0.05	NO	**YES** NS NS *p* < 0.05	**YES (kg/m^2^)/**NO *p* < 0.05 *p* < 0.05 *p* < 0.05	**YES** *p* < 0.05 *p* < 0.05 *p* < 0.05	**BW**: IG1: −7.91 ± 7.48 kg IG2: −15.45 ± 6.95 kg **BF**: IG1: −5.67 ± 8.05 kg IG1: −2.87 ± 6.01%IG2: −17.34 ± 6.5 kg IG2: −11.42 ± 6.10% **LM:** IG1: −2.29 ± 4.01 kg IG2: 2.31 ± 5.22 kg **BMI**: IG1: −2.62 ± 2.35 kg/m^2^ IG2: −5.54 ± 2.41 kg/m^2^ **WC**: IG1: −5.7 ± 6.37 cm IG2: −17.06 ± 11.38 cm
Yanovski et al., 2011 [61]	*n* = 100 IG1: 53 IG2: 47 Girls: 60% Age: 6–12 years old Population: North America (USA) Obesity/ Insulin resistance, source of diagnostic criteria: ≥ 95th Percentile /NE	6	**Dietary and physical exercise intervention, pharmacology** Within groups: IG1 (metformin) IG2 (placebo) Between groups	**YES (kg)** NS *p* < 0.05 *p* < 0.001	**YES (kg)** NS *p* < 0.05 ^(1)^ NS ^(2)^ *p* < 0.05	NO	NO	**YES (kg/m^2^ and *z*-score)/**NO *p* < 0.05 NS *p* < 0.05	**YES** NS *p* < 0.05 *p* < 0.05	**BW**: IG1: 1.47 kg (−0.31, 3.24) IG2: 4.85 kg (2.84, 6.85) **BF**: IG1: −0.48 kg (−0.8, 1.76) ^(1)^ IG2: −1.88 kg (0.44, 3.31) ^(1)^ IG1: −1.51 kg (−4.56, 1.54) ^(2)^ IG2: 1.81 kg (−1.64, 5.25) ^(2)^ **BMI**: IG1: −0.78 kg/m^2^ (−1.54, −0.01) IG2: 0.32 kg/m^2^ (−0.54, 1.18) IG1: −0.11 (−0.16, −0.05) (*z*-score) IG2: −0.04 (−0.1, 0.02) (*z*-score) **WC**: IG1: 1.84 cm (−1, 4.69) IG2: 4.38 cm (1.23, 7.53)
Clarson et al., 2009 [62]	*n* = 25 IG1: 11 IG2: 14 Boys: 56% Age: 10–16 years old Population: North America (Canada) Obesity/Insulin Resistance, source of diagnostic criteria: >95th Percentile /NE	6	**Physical exercise intervention, dietary education, pharmacology** Within groups: IG1 (metformin) IG2 (lifestyle alone) Between groups	NO	NO	NO	NO	**YES (kg/m^2^)** *p* < 0.05 NS *p* < 0.05	**YES** NS NS NS	**BMI**: IG1: −1.8 ± (0.8) kg/m^2^ IG2: 0.5 ± (0.3) kg/m^2^ **WC**: IG1 and IG2 NE (cm)
Atabek et al., 2008 [63]	*n* = 120 IG1: 90 IG2: 30 Girls: 50% Age: 9–17 years old Population: Europe (Turkey) Obesity/Hyperinsulinemia, source of diagnostic criteria: >95th Percentile /NE	6	**Dietary and physical exercise intervention pharmacology** Within groups: IG1 (metformin) IG2 (placebo) Between groups	**YES (kg)** *p* < 0.001 NS NE	NO	NO	NO	**YES (kg/m^2^)** *p* < 0.001 NS *p* < 0.01	NO	**BW**: IG1: −3.4 kg IG2: 3.6 kg **BMI**: IG1: −2.08 ± 2.32 kg/m^2^IG2: 0.65 ± 2.5 kg/m^2^
Love-Osborne et al., 2008 [64]	*n* = 64 IG1: 48 IG2: 16 Girls: 71% Age: 12–19 years old Population: North America (USA) Obesity/Insulin Resistance, source of diagnostic criteria: >95th Percentile /NE	6	**Dietary and physical exercise intervention, pharmacology** Within groups: IG1 (metformin) IG2 (placebo) Between groups	**YES (kg)** NE NE NS	NO	NO	NO	**YES (kg/m^2^)** NE NE NS	NO	**BW**: IG1 and IG2: NE (kg) **BMI**: IG1: −0.16 ± 1.89 kg/m^2^ IG2: 0.63 ± 1.29 kg/m^2^

Intervention groups (IG); kilograms (kg); centimeters (cm); body weight (BW); body fat (BF); Fat-free mass (FFM); lean mass (LM); Body Mass Index (BMI); medical Subject Heading (MeSH); metabolic syndrome (MS); study of the analyzed anthropometric parameter (YES); The article does not include its study (NO); not significant (NS); there is a variation of the anthropometric parameter, but this information is not available in the clinical trial evaluated (NE); percentage (%); waist circumference (WC); ^(1)^ BF by air displacement plethysmography (kg); ^(2)^ BF by DEXA (kg); ^(3)^ Median (IQR).

## Supplementary Materials

The following are available online at http://www.mdpi.com/2072-6643/10/7/878/s1, Table S1: PRISMA 2009 Checklist ^(63)^.

## References

[1] Diem G., Brownson R.C., Grabauskas V., Shatchkute A., Stachenko S., Diem G., Brownson R.C., Grabauskas V., Shatchkute A., Stachenko S. (2016). Prevention and control of noncommunicable diseases through evidence-based public health: Implementing the NCD 2020 Action Plan. Glob. Health Promot..

[2] Riley L., Guthold R., Cowan M., Savin S., Bhatti L., Timothy Armstrong T., Bonita R. (2016). The World Health Organization STEPwise Approach to Noncommunicable Disease Risk-Factor Surveillance: Methods, Challenges, and Opportunities. Am. J. Public Health.

[3] Rodríguez-Monforte M., Sánchez E., Barrio F., Costa B., Flores-Mateo G. (2016). Metabolic syndrome and dietary patterns: A systematic review and meta-analysis of observational studies. Eur. J. Nutr..

[4] Yamaoka K., Tango T. (2012). Effects of lifestyle modification on metabolic syndrome: A systematic review and meta-analysis. BMC Med..

[5] Grundy S.M. (2016). Metabolic syndrome update. Trends Cardiovasc. Med..

[6] Han T.S., Lean M.E. (2016). A clinical perspective of obesity, metabolic syndrome and cardiovascular disease. JRSM Cardiovasc. Dis..

[7] Dulloo A.G., Montani J.P. (2015). Pathways from dieting to weight regain, to obesity and to the metabolic syndrome: An overview. Obes. Rev..

[8] Hong A.R., Lim S. (2015). Clinical characteristics of metabolic syndrome in Korea, and its comparison with other Asian countries. J. Diabetes Investig..

[9] Calton E.K., James A.P., Pannu P.K., Soares M.J. (2014). Certain dietary patterns are beneficial for the metabolic syndrome: Reviewing the evidence. Nutr. Res..

[10] Kaur J. (2014). A Comprehensive Review on Metabolic Syndrome. Cardiol. Res. Pract..

[11] Weiss R., Bremer A.A., Lustig R.H. (2013). What is metabolic syndrome, and why are children getting it?. Ann. N. Y. Acad. Sci..

[12] Jain V.K., Badjatya V., Nema R.K. (2013). A review on the metabolic syndrome: Plethora of disease. Adv. Pharmacol. Toxicol..

[13] Nelson R.A., Bremer A.A. (2010). Insulin Resistance and Metabolic Syndrome in the Pediatric Population. Metab. Syndr. Relat. Disord..

[14] Halpern A., Mancini M.C., Magalhães M.E.C., Fisberg M., Radominski R., Bertolami M.C., Adriana Bertolami A., de Melo M.E., Zanella M.T., Queiroz M.S. (2010). Metabolic syndrome, dyslipidemia, hypertension and type 2 diabetes in youth: From diagnosis to treatment. Diabetol. Metab. Syndr..

[15] Fleischman A., Rhodes E.T. (2009). Management of obesity, insulin resistance and type 2 diabetes in children: Consensus and controversy. Diabetes Metab. Syndr. Obes..

[16] Hanefeld M., Pistrosch F., Bornstein S.R., Birkenfeld A.L. (2016). The metabolic vascular syndrome—Guide to an individualized treatment. Rev. Endocr. Metab. Disord..

[17] Pucci G., Alcidi R., Tap L., Battista F., Mattace-Raso F., Schillaci G. (2017). Sex-and gender-related prevalence, cardiovascular risk and therapeutic approach in metabolic syndrome: A review of the literature. Pharmacol. Res..

[18] Graf C., Ferrari N. (2016). Metabolic Syndrome in Children and Adolescents. Visc. Med..

[19] Kelishadi R., Hovsepian S., Djalalinia S., Qorbani M. (2016). A systematic review on the prevalence of metabolic syndrome in Iranian children and adolescents. J. Res. Med. Sci..

[20] Oliveros E., Somers V.K., Sochor O., Goel K., Lopez-Jimenez F. (2014). The concept of normal weight obesity. Prog. Cardiovasc. Dis..

[21] Pacifico L., Anania C., Martino F., Poggiogalle E., Chiarelli F., Arca M., Chiesa C. (2011). Management of metabolic syndrome in children and adolescents. Nutr. Metab. Cardiovasc. Dis..

[22] Huang T.T.K., Ball G.D.C., Franks P.W. (2007). Metabolic syndrome in youth: Current issues and Challenges. Appl. Physiol. Nutr. Metab..

[23] Titmuss A.T., Srinivasan S. (2016). Metabolic syndrome in children and adolescents: Old concepts in a young population. J. Paediatr. Child Health.

[24] Tavares Giannini D., Caetano Kuschnir M.C., Szklo M. (2014). Metabolic Syndrome in Overweight and Obese Adolescents: A Comparison of Two Different Diagnostic Criteria. Ann. Nutr. Metab..

[25] Haemer M.A., Grow H.M., Fernandez C., Lukasiewicz G.J., Rhodes E.T., Shaffer L.A., Sweeney B., Woolford S.J., Estrada E. (2014). Addressing prediabetes in childhood obesity treatment programs: Support from research and current practice. Child. Obes..

[26] American Diabetes Association (ADA) (2014). Diagnosis and Classification of Diabetes Mellitus. Diabetes Care..

[27] Hoelscher D.M., Kirk S., Ritchie L., Cunningham-Sabo L. (2013). Academy Positions Committee. Position of the Academy of Nutrition and Dietetics: Interventions for the Prevention and Treatment of Pediatric Overweight and Obesity. J. Acad. Nutr. Dietet..

[28] Seagle H.M., Strain G.W., Makris A., Reeves R.S. (2009). American Dietetic Association. Position of the American Dietetic Association: Weight management. J. Am. Dietet. Assoc..

[29] Fitch A., Fox C., Bauerly K., Gross A., Heim C., Judge-Dietz J., Kaufman T., Krych E., Kumar S., Landin D. Prevention and Management of Obesity for Children and Adolescents. https://www.ohcoop.org/wp-content/uploads/Clinical-Guidelines-Prevention-and-Management-Obesity-in-Children-and-Adolscent.pdf.

[30] Copeland K.C., Silverstein J., Moore K.R., Prazar G.E., Raymer T., Shiffman R.N., Springer S.C., Thaker V.V., Anderson M., Spann S.J. (2013). Management of newly diagnosed type 2 Diabetes Mellitus (T2DM) in children and adolescents. Pediatrics.

[31] American Diabetes Association (ADA) (2017). 4. Lifestyle Management. Diabetes Care.

[32] O’Connor E.A., Evans C.V., Burda B.U., Walsh E.S., Eder M., Lozano P. (2017). Screening for obesity and intervention for weight management in children and adolescents: Evidence report and systematic review for the US Preventive Services Task Force. JAMA.

[33] (2016). Consideration of the Evidence on Childhood Obesity for the Commission on Ending Childhood Obesity: Report of the ad Hoc Working Group on Science and Evidence for Ending Childhood Obesity, Geneva, Switzerland. http://apps.who.int/iris/bitstream/10665/206549/1/9789241565332_eng.pdf?ua=1.

[34] Onge E.S., Miller S.A., Motycka C., DeBerry A. (2015). A review of the treatment of type 2 diabetes in children. J. Pediatr. Pharmacol. Ther..

[35] National Health and Medical Research Council (NHMRC) Clinical Practice Guidelines for the Management of Overweight and Obesity in Adults, Adolescents and Children in Australia. https://www.nhmrc.gov.au/guidelines-publications/n57.

[36] Logue J. (2010). Management of Obesity. A National Clinical Guideline.

[37] August G.P., Caprio S., Fennoy I., Freemark M., Kaufman F.R., Lustig R.H., Silverstein J.H., Speiser P.W., Styne D.M., Montori V.M. (2008). Prevention and treatment of pediatric obesity: An endocrine society clinical practice guideline based on expert opinion. J. Clin. Endocrinol. Metab..

[38] Reig M., Rizo M.M., Cortés E. (2015). Predictors of weight loss and fat in the dietary management: Sex, age, BMI and consultin assistance. Nutr. Hosp..

[39] Gutiérrez A., Reig M., Rizo M., Cortés E., Mur N., Aguilar M.I. (2014). Measurement units used in treatments to reduce weight and obesity. Systematic review. Nutr. Hosp..

[40] McCarthy H.D., Samani-Radia D., Jebb S.A., Prentice A.M. (2014). Skeletal muscle mass reference curves for children and adolescents. Ped. Obes..

[41] McCarthy H.D. (2014). Measuring growth and obesity across childhood and adolescence. Proc. Nutr. Soc..

[42] Oguoma V.M., Nwose E.U., Richards R.S. (2015). Prevalence of cardio-metabolic syndrome in Nigeria: A systematic review. Public Health.

[43] Duprez D., Toleuova A. (2013). Prehypertension and the cardiometabolic syndrome: Pathological and clinical consequences. Expert Rev. Cardiovasc. Ther..

[44] Stoner L., Rowlands D., Morrison A., Credeur D., Hamlin M., Gaffney K., Lambrick D., Matheson A. (2016). Efficacy of Exercise Intervention for Weight Loss in Overweight and Obese Adolescents: Meta-Analysis and Implications. Sports Med..

[45] Ho M., Garnett S.P., Baur L., Burrows T., Stewart L., Neve M., Collins C. (2012). Effectiveness of lifestyle interventions in child obesity: Systematic review with meta-analysis. Pediatrics.

[46] Martínez-González M.Á., Martín-Calvo N. (2013). The major European dietary patterns and metabolic syndrome. Rev. Endocr. Metab. Disord..

[47] Quinn S.M., Baur L.A., Garnett S.P., Cowell C.T. (2010). Treatment of clinical insulin resistance in children: A systematic review. Obes. Rev..

[48] Khokhar A., Umpaichitra V., Chin V.L., Perez-Colon S. (2017). Metformin Use in Children and Adolescents with Prediabetes. Pediatr. Clin. N. Am..

[49] Ho M., Garnett S.P., Baur L.A. (2014). Childhood Obesity and Insulin Resistance: How Should It Be Managed?. Curr. Treat. Opt. Cardiovasc. Med..

[50] American Diabetes Association (ADA) (2017). 12. Children and Adolescents. Diabetes Care.

[51] Rodd C., Metzger D.L., Sharma A. (2014). Canadian Pediatric Endocrine Group (CPEG) Working Committee for National Growth Charts. Extending World Health Organization weight-for-age reference curves to older children. BMC Pediatr..

[52] Moher D., Liberati A., Tetzlaff J., Altman D.G. (2009). Preferred reporting items for systematic reviews and meta-analyses: The PRISMA statement. Ann. Inter. Med..

[53] Hutton B., Salanti G., Caldwell D.M., Chaimani A., Schmid C.H., Cameron C., Ioannidis J.P.A., Straus S., Thorlund K., Jansen J.P. (2015). The PRISMA Extension Statement for Reporting of Systematic Reviews Incorporating Network Meta-analyses of Health Care Interventions: Checklist and Explanations. Ann. Intern. Med..

[54] Liberati A., Altman D.G., Tetzlaff J., Mulrow C., Gøtzsche P.C., Ioannidis J.P.A., Clarke M., Devereaux P.J., Kleijnen J., Moher D. (2009). The PRISMA statement for reporting systematic reviews and meta-analyses of studies that evaluate health care interventions: Explanation and elaboration. PLoS Med..

[55] Wiesman F., Hasman A., van den Herik H.J. (1997). Information retrieval: An overview of system characteristics. Int. J. Med. Inform..

[56] de Mello M.T., de Piano A., Carnier J., Sanches P.D.L., Corrêa F.A., Tock L., Ernandes R.M.Y., Tufik S., Dâmaso A.R. (2011). Long-term effects of aerobic plus resistance training on the metabolic syndrome and adiponectinemia in obese adolescents. J. Clin. Hypertens..

[57] Armeno M.L., Krochik A.G., Mazza C.S. (2011). Evaluation of two dietary treatments in obese hyperinsulinemic adolescents. J. Pediatr. Endocrinol. Metab..

[58] Van der Aa M.P., Elst M.A.J., Van De Garde E.M.W., Van Mil E.G.A.H., Knibbe C.A.J., Van der Vorst M.M.J. (2016). Long-term treatment with metformin in obese, insulin-resistant adolescents: Results of a randomized double-blinded placebo controlled trial. Nutr. Diabetes.

[59] Garnett S.P., Gow M., Ho M., Baur L.A., Noakes M., Woodhead H.J., Broderick C.R., Burrell S., Chisholm K., Halim J. (2013). Optimal macronutrient content of the diet for adolescents with prediabetes; RESIST a randomised control trial. J. Clin. Endocrinol. Metab..

[60] Gómez-Díaz R.A., Talavera J.O., Pool E.C., Ortiz-Navarrete F.V., Solórzano-Santos F., Mondragón-González R., Valladares-Salgado A., Cruz M., Aguilar-Salinas C.A., Wacher N.H. (2012). Metformin decreases plasma resistin concentrations in pediatric patients with impaired glucose tolerance: A placebo-controlled randomized clinical trial. Metabolism.

[61] Yanovski J.A., Krakoff J., Salaita C.G., McDuffie J.R., Kozlosky M., Sebring N.G., Reynolds J.C., Brady S.M., Calis K.A. (2011). Effects of metformin on body weight and body composition in obese insulin-resistant children: A randomized clinical trial. Diabetes.

[62] Clarson C.L., Mahmud F.H., Baker J.E., Clark H.E., Mckay W.M., Schauteet V.D., Hill D.J. (2009). Metformin in combination with structured lifestyle intervention improved body mass index in obese adolescents, but did not improve insulin resistance. Endocrine.

[63] Atabek M.E., Pirgon O. (2008). Use of metformin in obese adolescents with hyperinsulinemia: A 6-month, randomized, double-blind, placebo-controlled clinical trial. J. Pediatr. Endocrinol. Metab..

[64] Love-Osborne K., Sheeder J., Zeitler P. (2008). Addition of metformin to a lifestyle modification program in adolescents with insulin resistance. J. Pediatr..

[65] Mead E., Brown T., Rees K., Azevedo L.B., Whittaker V., Jones D., Olajide J., Mainardi G.M., Corpeleijn E., O’Malley C. (2017). Diet, physical activity and behavioural interventions for the treatment of overweight or obese children from the age of 6 to 11 years. Cochrane Database Syst. Rev..

[66] García-Hermoso A., Sánchez-López M., Martínez-Vizcaíno V. (2015). Effects of Aerobic Plus Resistance Exercise on Body Composition Related Variables in Pediatric Obesity: A Systematic Review and Meta-Analysis of Randomized Controlled Trials. Pediatr. Exerc. Sci..

[67] Ho M., Garnett S.P., Baur L.A., Burrows T., Stewart L., Neve M., Collins C. (2013). Impact of dietary and exercise interventions on weight change and metabolic outcomes in obese children and adolescents: A systematic review and meta-analysis of randomized trials. JAMA Pediatr..

[68] Larsen T.M., Dalskov S., Van Baak M., Jebb S., Kafatos A., Pfeiffer A., Martinez J.A., Handjieva-Darlenska T., Kunešová M., Holst C. (2010). The Diet, Obesity and Genes (Diogenes) Dietary Study in eight European countries – a comprehensive design for long-term intervention. Obes. Rev..

[69] Whitlock E.P., O’Connor E.A., Williams S.B., Beil T.L., Lutz K.W. (2010). Effectiveness of Weight Management Interventions in Children: A Targeted Systematic Review for the USPSTF. Pediatrics.

[70] Gow M.L., Garnett S.P., Baur L.A., Lister N.B. (2016). The Effectiveness of Different Diet Strategies to Reduce Type 2 Diabetes Risk in Youth. Nutrients.

[71] Kroeger C.M., Hoddy K.K., Varady K.A. (2014). Impact of weight regain on metabolic disease risk: A review of human trials. J. Obes..

[72] Moher D., Hopewell S., Schulz K.F., Montori V., Gøtzsche P.C., Devereaux P.J., Elbourne D., Egger M., Altman D.G. (2012). CONSORT 2010 explanation and elaboration: Updated guidelines for reporting parallel group randomised trials. Int. J. Surg..

[73] Argimon J.M., Jimenéz J. (2013). Métodos de investigación clínica y epidemiológica. Elsevier.

[74] Reig M., Rizo M.M., Cortés E. (2015). Indicators of success in the dietary management of overweight and obesity: Weight, body fat loss and quality. Nutr. Hosp..

[75] Millstein R.A. (2014). Measuring Outcomes in Adult Weight Loss Studies That Include Diet and Physical Activity: A Systematic Review. J. Nutr. Metab..

[76] McCarthy H.D., Cole T.J., Fry T., Jebb S.A., Prentice A.M. (2006). Body fat reference curves for children. Int. J. Obes. (Lond.).

[77] Reig M.R., Castell E.C., Baeza M.R., Hervás A.G. (2015). The variability in adherence to dietary treatment and quality of weight loss: Overweight and obesity. Nutr. Hosp..

[78] Stubbs J., Whybrow S., Teixeira P., Blundell J., Lawton C., Westenhoefer J., Engel D., Shepherd R., Mcconnon Á., Gilbert P. (2011). Problems in identifying predictors and correlates of weight loss and maintenance: Implications for weight control therapies based on behaviour change. Obes. Rev..

[79] Desbrow B., McCormack J., Burke L.M., Cox G.R., Fallon K., Hislop M., Logan R., Marino N., Sawyer S.M., Shaw G. (2014). Sports Dietitians Australia Position Statement: Sports Nutrition for the Adolescent Athlete. Int. J. Sport Nutr. Exerc. Metab..

[80] Jeukendrup A.E. (2011). Nutrition for endurance sports: Marathon, triathlon, and road cycling. J. Sports Sci..

[81] Jensen M.D., Ryan D.H. (2014). 2013 AHA/ACC/TOS. Guideline for management of overweight and obesity in adults. J. Am. Coll. Cardiol..

[82] Garber C.E., Blissmer B., Deschenes M.R., Franklin B.A., Lamonte M.J., Lee I.M., Nieman D.C., Swain D.P. (2011). American College of Sports Medicine position stand (ACSM). Quantity and quality of exercise for developing and maintaining cardiorespiratory, musculoskeletal, and neuromotor fitness in apparently healthy adults: Guidance for prescribing exercise. Med. Sci. Sports Exerc..

[83] Pieles G.E., Horn R., Williams C.A., Stuart A.G. (2014). Paediatric exercise training in prevention and treatment. Arch. Dis. Child..

[84] Mielgo-Ayuso J., Aparicio-Ugarriza R., Castillo A., Ruiz E., Avila J.M., Aranceta-Bartrina J., Angel Gil A., Ortega R.M., Serra-Majem L., Varela-Moreiras G. (2017). Sedentary behavior among Spanish children and adolescents: Findings from the ANIBES study. BMC Public Health.

[85] Ravussin E., Ryan D. (2016). Energy Expenditure and Weight Control: Is the Biggest Loser the Best Loser?. Obesity.

[86] Ryan D., Heaner M. (2014). Guidelines (2013) for managing overweight and obesity in adults: Preface to the full report. Obesity (Silver Spring).

[87] Cefalu W.T., Bray G.A., Home P.D., Garvey W.T., Klein S., Pi-Sunyer F.X., Hu F.B., Raz I., Gaal V.L., Wolfe B.M. (2015). Advances in the Science, Treatment, and Prevention of the Disease of Obesity: Ref lections From a Diabetes Care Editors’ Expert Forum. Diabetes Care.

[88] Delahanty L.M. (2010). An expanded role for dietitians in maximising retention in nutrition and lifestyle intervention trials: Implications for clinical practice. J. Hum. Nutr. Diet..

[89] Spear B.A., Barlow S.E., Ervin C., Ludwig D.S., Saelens B.E., Schetzina K.E., Taveras E.M. (2007). Recommendations for Treatment of Child and Adolescent Overweight and Obesity. Pediatrics.

[90] Reig M. (2015). The management of qualitative and quantitative dietary treatment for overweight and obesity: Methodology and a new perspective on individualised assessment. Nutr. Hosp..

[91] Apovian C.M., Garvey W.T., Ryan D.H. (2015). Challenging Obesity: Patient, Provider, and Expert Perspectives on the Roles of Available and Emerging Nonsurgical Therapies. Obesity.

[92] Quiles P., Reig M. (2015). Glycemic control through physical exercise in type 2 diabetes systematic review. Nutr. Hosp..

[93] Albert Pérez E.J., Reig García-Galbis M. (2015). Effects of green tea on the nutritional status of the exercise. Nutr. Hosp..

